# Prognosis and predictive factors in pediatric IgA nephropathy

**DOI:** 10.1007/s00467-025-06988-8

**Published:** 2025-11-13

**Authors:** Wenpei Liang, Yonghua He, Xueqing Ma, Panpan Shao, Ling Guo, Jianhua Zhou, Yu Zhang, Huiqing Yuan, Liru Qiu

**Affiliations:** 1 Department of Pediatrics, Tongji Hospital, Tongji Medical College, Huazhong University of Science and Technology, 1095 Jiefang Avenue, Qiaokou District, Wuhan, 430030 China; 2 Hubei Provincial Key Laboratory of Pediatric Genetic Metabolic and Endocrine Rare Diseases, Wuhan, 430030 China; 3 Hubei Provincial Clinical Research Center for Childrens Growth and Development and Metabolic Diseases, Wuhan, 430030 China

**Keywords:** IgA nephropathy, Children, Poor prognosis, Kidney complete remission

## Abstract

**Background:**

IgA nephropathy (IgAN) is a common glomerular disease in children that may progress to chronic kidney disease (CKD) and kidney failure. Identifying reliable prognostic markers is important for guiding clinical management. This study investigated independent predictors of poor prognosis in pediatric IgAN and their impact on eGFR slope, as well as the dynamic patterns of complete kidney remission and relapse, predictive factors associated with remission, and the influence of remission on poor prognosis.

**Methods:**

A retrospective cohort study of 224 children (aged 3–18 years) with biopsy-proven IgAN and ≥ 3 years of follow-up was enrolled. Poor prognosis was defined as persistent eGFR < 90 mL/min/1.73 m^2^ for ≥ 3 months. Multivariate logistic regression, receiver operating characteristic (ROC) curve analysis, and linear mixed-effects model were used to identify predictive factors and evaluate eGFR slope. Complete kidney remission was defined as the absence of hematuria and proteinuria with eGFR ≥ 90 mL/min/1.73 m^2^ for ≥ 1 year. Cumulative incidence function and Fine-Gray competing risk regression model were applied to analyze remission dynamic patterns.

**Results:**

Over a median follow-up of 5.41 years, 12.05% reached the poor prognosis. Independent risk factors included male sex, older age at biopsy, Oxford classification E1 and S1 lesions, absence of gross hematuria, and no remission of proteinuria during follow-up, while higher birth weight was protective. eGFR slope was influenced by age at biopsy, gross hematuria and proteinuria remission status during follow-up. Complete kidney remission occurred in 70.98%, with a recurrence rate of 60.38%. Predictors of complete kidney remission included antecedent infection, kidney IgM deposition of 2 + , elevated serum IgM, increased pathological casts count, and prednisone treatment, while prolonged disease duration before biopsy and higher tubular casts count were risk factors. Group comparisons and logistic regression analysis showed no significant associations between kidney remission and poor.

**Conclusions:**

Pediatric IgAN shows slow disease progression over 5 years. Despite high complete kidney remission rates, recurrence remains common. These findings support extended follow-up and improved predictive models.

**Graphical abstract:**

**Figure:**
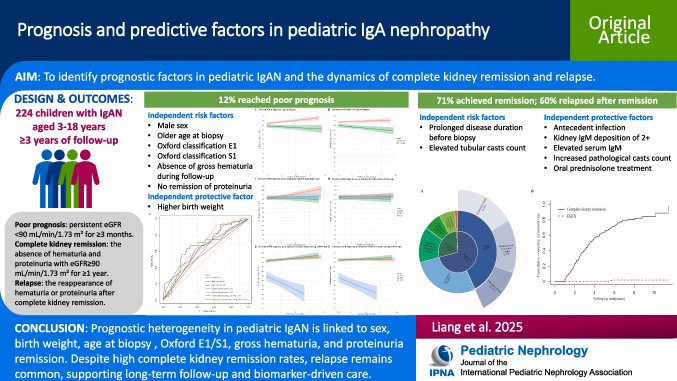
A higher resolution version of the Graphical abstract is available as Supplementary information

**Supplementary Information:**

The online version contains supplementary material available at 10.1007/s00467-025-06988-8.

## Introduction

IgA nephropathy (IgAN) is the most prevalent primary glomerulonephritis globally, with incidence rates ranging from 5.4 to 105 cases per million person-years. Its prevalence varies substantially due to differences in geographic, environmental, socioeconomic, and genetic factors, as well as healthcare access, biopsy practices, and urinalysis screening policies [1–3]. While IgAN can occur at any age, it is more frequently diagnosed in children and young adults than the elderly. Pediatric and adult IgAN differ in clinical features, pathological patterns, disease progression, and treatment response, raising ongoing debate as to whether they represent distinct disease entities or age-related phenotypes along a shared continuum [4, 5]. In adults, IgAN typically progresses gradually, with 25%–30% reaching kidney failure within 10 years and up to 40% within 20 years after diagnosis [6]. In contrast, children often follow a more favorable short-term course, though long-term prognosis remain concerning. While some achieve spontaneous remission, 5%–10% may progress rapidly to kidney failure in childhood [7]. Most pediatric cases show slow deterioration during childhood, with 20%–30% progressing to kidney failure over 20 years [5, 7–10]. Given the extended life expectancy of children, the cumulative disease burden ultimately parallels that of adults, underscoring the need for early intervention and long-term monitoring. Although kidney biopsy is critical for diagnosis and management, its invasiveness limits repeat application in children. Therefore, noninvasive biomarkers are urgently needed to enable dynamic monitoring and early risk stratification in pediatric IgAN.

Current studies have predominantly focused on identifying predictive factors, yet existing international models show limited performance in Chinese cohorts [11–13]. Additionally, no standardized criteria exist for assessing complete kidney remission. The 2024 International Pediatric Nephrology Association (IPNA) guidelines define pediatric complete kidney remission as the absence of hematuria (urinary red blood cells < 5/HPF), proteinuria (urinary protein-to-creatinine ratio [UPCR] < 200 mg/g) and estimated glomerular filtration rate (eGFR) ≥ 90 mL/min/1.73 m^2^ for ≥ 1 month. However, these criteria remain unvalidated in Asian children, and studies on predictive factors remain scarce [14]. To address this gap, we retrospectively analyzed long-term follow-up data from pediatric patients with IgAN at our center. We aimed to identify independent predictors of poor prognosis and to assess their impact on eGFR slope. Additionally, we analyzed the dynamic patterns of complete kidney remission and relapse, identified predictive factors associated with complete kidney remission, and evaluated the influence of kidney remission on poor prognosis. These findings may provide evidence to support precision management and pediatric-specific risk assessment strategies for IgAN.

## Materials and methods

### Study design and participants

This study included pediatric patients diagnosed with IgAN via initial kidney biopsy at the Tongji Hospital from January 2012 to December 2020. Inclusion criteria included: (1) age ≥ 3 and < 18 years at biopsy; (2) follow-up duration ≥ 3 years; (3) complete baseline pathology and laboratory data; (4) at least three eGFR measurements during follow-up; and (5) urinalysis and UPCR measurements at intervals of ≤ 6 months. Exclusion criteria included: (1) secondary IgAN (e.g., IgA vasculitis nephritis, lupus nephritis, HBV-associated nephritis); (2) < 8 glomeruli on light microscopy; (3) coexisting kidney diseases (e.g., Alport syndrome, membranous nephropathy, interstitial nephritis); (4) kidney failure at presentation; (5) post-transplant IgAN; and (6) family history of kidney failure. The study was approved by the Tongji Hospital Ethics Committee (Approval No. TJ-IRB202412138), with informed consent waived due to anonymized retrospective data.

### Data collection

The study used retrospective medical records, with follow-up data updated through September 2024. Study data were categorized into four domains: (1) baseline characteristics; (2) kidney pathology scored using the Oxford MEST-C classification, in which mesangial hypercellularity (M) is scored M0 (< 50% of glomeruli involved) or M1 (≥ 50%), endocapillary hypercellularity (E) as E0 (absent) or E1 (present), segmental glomerulosclerosis (S) as S0 (absent) or S1 (present), tubular atrophy/interstitial fibrosis (T) as T0 (0–25%), T1 (26–50%), or T2 (> 50%), and crescents (C) as C0 (0%), C1 (1–24%), or C2 (≥ 25% of glomeruli). All pathological assessments were independently performed by two senior kidney pathologists blinded to clinical information, with discrepancies resolved by consensus or adjudicated by a third expert; (3) clinical parameters at biopsy; and (4) follow-up information, including therapeutic interventions (supportive treatment, renin-angiotensin system blockers [RASB], oral prednisolone [PDN], other immunosuppressants [IS], and tonsillectomy performed) and kidney remission, etc.

### Definitions

eGFR was calculated using the serum creatinine (SCr)–based equation from the European Kidney Function Consortium (EKFC), which overcomes the problem of implausible changes in GFR during the transition from adolescence to adulthood, thereby ensuring continuity and stability of eGFR in longitudinal follow-up [15, 16]. Family history of kidney disease was defined as a diagnosis of IgAN, nephrotic syndrome, glomerulonephritis, kidney failure, or abnormal urinalysis indicative of kidney disease. Antecedent infection was defined as a clinically documented infection within 4 weeks prior to disease onset or kidney biopsy, confirmed by medical records and/or laboratory evidence (e.g., upper respiratory or gastrointestinal tract infection). Time-averaged hematuria (TA-H) and time-averaged UPCR (TA-UPCR) were calculated as the area under the time-series curve (AUC), divided by total follow-up duration to reflect cumulative exposure. Gross hematuria status during follow-up was categorized as recurrent (RGH, ≥ 2 episodes), isolated (IGH, 1 episode), or none (NGH). Proteinuria remission status during follow-up was categorized as complete (CRP, TA-UPCR < 200 µg/mg), partial (PRP, TA-UPCR 200–1999 µg/mg), or no remission (NRP, TA-UPCR ≥ 2000 µg/mg).

### Outcomes

#### Poor prognosis

We analyzed two outcome constructs. (1) Hard outcomes recorded descriptively: kidney failure and/or a ≥ 30% reduction in eGFR from baseline. (2) The binary endpoint used for between-group comparisons and multivariable logistic regression was defined as sustained eGFR < 90 mL/min/1.73 m^2^ for ≥ 3 months, defining the endpoint and non-endpoint groups. Additionally, annual decline in eGFR (eGFR slope) was evaluated as a longitudinal outcome in two ways: the overall eGFR slope, calculated using all eGFR values from baseline to the last follow-up, and the chronic eGFR slope, calculated after excluding the first 3 months to minimize the confounding effects of acute-phase treatments.

#### Complete kidney remission

Complete kidney remission was defined as the absence of both hematuria and proteinuria for ≥ 1 year, with eGFR ≥ 90 mL/min/1.73 m^2^. Relapse referred to the recurrence of hematuria or proteinuria after remission. According to kidney remission, patients were classified into three groups: (1) complete remission without relapse, (2) complete remission with relapse, and (3) no remission or progression to kidney failure.

### Statistical analysis

Statistical analyses were conducted using SPSS 25.0 and R 4.4.2. Continuous variables were presented as mean ± SD or median (IQR), and compared using the t-test or Mann–Whitney U test. Categorical variables were expressed as counts (%) and compared using the χ^2^ test or Mann–Whitney U test, as appropriate. Two-tailed *p*-value < 0.05 was considered statistically significant.

Univariate logistic regression identified predictive factors for poor prognosis. Variables with *p*-value < 0.05 in intergroup comparisons or clinical relevance were included. Variables with *p*-value < 0.1 underwent collinearity checks (VIF > 5 excluded). Multivariate logistic regression was performed via stepwise selection. Continuous variables were dichotomized based on optimal ROC-derived cutoffs (Youden index).

Linear mixed models (LMMs) with random intercepts and slopes were used to assess time-dependent predictors of eGFR slope. Model 1 included time and group; Model 2 added their interaction. Interaction significance was evaluated by likelihood ratio test (LRT), Akaike Information Criterion (AIC), and Bayesian Information Criterion (BIC). Significant interaction (LRT *p*-value < 0.05 with lower AIC/BIC) indicated time-dependent effects.

For competing risks analysis of kidney failure, kidney outcomes were classified as 0 = censored, 1 = complete kidney remission, and 2 = kidney failure. The cumulative incidence of complete kidney remission was estimated via cumulative incidence function (CIF), with variables (*p*-value < 0.05) adjusted using the Fine–Gray model. Multivariate logistic regression assessed associations between kidney remission and poor prognosis, adjusting for sex, birth weight, age at biopsy, Oxford classification E and S lesions, gross hematuria status during follow-up, TA-H, and TA-UPCR.

## Results

### Baseline clinicopathological characteristics and overall outcomes

A total of 224 children with biopsy-proven IgAN were included. The enrollment flowchart is shown in Supplementary Fig. 1. Key clinical, pathological, and follow-up data are shown in Table 1, with additional data provided in Supplementary Table 1. The overall male-to-female ratio was approximately 3:1. The median age at biopsy was 9.68 (7.96, 12.02) years, and the median follow-up duration was 5.41 (3.96, 6.78) years. According to the Oxford classification, the proportions of M1, E1, S1, T1–2, and C1–2 lesions were 29.46%, 31.25%, 66.96%, 34.82%, and 60.71%, respectively. The median eGFR at biopsy was 107.00 (83.38, 113.40) mL/min/1.73 m^2^. The median TA-H and TA-UPCR were 46.53 (19.00, 102.35)/μL and 96.44 (34.57, 243.35) μg/mg, respectively. At last follow-up, the median eGFR was 109.05 (100.80–113.00) mL/min/1.73 m^2^; 2.23% had ≥ 30% eGFR decline, 1.34% had ≥ 50% eGFR decline, and 1.34% progressed to kidney failure.

**Table 1:** Summary of clinical, pathological, and follow-up data in pediatric patients with IgA nephropathy

Variables	Total group (*n* = 224)	Non-endpoint group (*n* = 197)	Endpoint group (*n* = 27)	*p*-value
Baseline characteristics				
Sex, male, *n*(%)	166 (74.11)	141 (71.57)	25 (92.59)	0.019
Gestational age, *n*(%)				0.039
Preterm birth	7 (3.12)	4 (2.03)	3 (11.11)	
Term birth	217 (96.88)	193 (97.97)	24 (88.89)	
Birth weight (kg)	3.27 ± 0.46	3.30 ± 0.44	3.07 ± 0.52	0.013
Family history of kidney disease, *n*(%)	32 (14.29)	23 (11.68)	9 (33.33)	0.006
Age at onset (years)	9.36 (7.59, 11.48)	9.15 (7.46, 11.02)	11.16 (8.37, 12.43)	0.027
Age at biopsy (years)	9.68 (7.96, 12.02)	9.50 (7.84, 11.48)	12.06 (9.27, 12.50)	0.004
Disease duration before biopsy (months)	1.07 (0.60, 3.33)	1.07 (0.60, 3.13)	2.13 (0.81, 5.63)	0.058
Antecedent infection, *n*(%)	100 (44.64)	87 (44.16)	13 (48.15)	0.696
Height (cm)	139.72 ± 15.87	138.46 ± 15.67	148.91 ± 14.52	0.001
Weight (kg)	32.75 (26.15, 41.15)	31.30 (25.00, 40.00)	42.80 (33.20, 53.50)	< 0.001
BMI (kg/m^2^)	16.79 (15.07, 19.64)	16.57 (14.92, 18.94)	19.38 (16.50, 22.03)	0.002
SBP (mmHg)	105.85 ± 12.45	104.87 ± 11.46	112.96 ± 16.69	0.021
DBP (mmHg)	69.00 (63.00, 76.00)	69.00 (62.00, 75.00)	72.00 (68.50, 79.00)	0.020
MAP (mmHg)	81.33 (75.67, 87.33)	80.67 (75.33, 86.67)	85.67 (80.00, 92.66)	0.007
Kidney pathology				
Oxford classification, *n*(%)				
M1	66 (29.46)	57 (28.93)	9 (33.33)	0.638
E1	70 (31.25)	56 (28.43)	14 (51.85)	0.014
S1	150 (66.96)	125 (63.45)	25 (92.59)	0.003
T1	69 (30.80)	61 (30.96)	8 (29.63)	0.633
T2	9 (4.02)	7 (3.55)	2 (7.41)	
C1	110 (49.11)	97 (49.24)	13 (48.15)	0.853
C2	26 (11.61)	22 (11.17)	4 (14.81)	
Clinical parameters at biopsy				
Hematuria status, *n*(%)				1.000
No hematuria	9 (4.02)	8 (4.06)	1 (3.70)	
Isolated hematuria	8 (3.57)	7 (3.55)	1 (3.70)	
Hematuria + proteinuria	207 (92.41)	182 (92.39)	25 (92.59)	
Proteinuria status, *n*(%)				0.986
No proteinuria	8 (3.57)	7 (3.55)	1 (3.70)	
Non-nephrotic syndrome level	136 (60.71)	120 (60.91)	16 (59.26)	
Nephrotic syndrome level	80 (35.71)	70 (35.53)	10 (37.04)	
UACR (μg/mg)	513.20 (160.23, 2116.09)	512.70 (174.88, 2067.10)	609.20 (107.90, 2498.70)	0.887
UPCR (μg/mg)	784.13 (330.06, 2780.43)	779.28 (350.72, 2777.80)	788.97 (256.69, 3242.66)	0.660
24 h-Ualb (mg/d/1.73 m^2^)	560.64 (121.35, 2152.01)	560.26 (122.42, 2115.73)	1134.32 (128.60, 2576.74)	0.798
24 h-UTP (mg/d/1.73 m^2^)	828.02 (320.91, 2747.09)	817.5 (322.00, 2719.47)	1356.91 (319.85, 3610.75)	0.899
eGFR at biopsy (ml/min/1.73 m^2^)	107 (83.38, 113.40)	107.5 (87.00, 113.80)	95.4 (69.20, 110.25)	0.042
Scr (µmol/L)	45.00 (38.00, 60.25)	44.00 (37.00, 59.00)	58.00 (45.50, 82.00)	< 0.001
CysC (mg/L)	0.93 (0.84, 1.06)	0.92 (0.83, 1.04)	1.03 (0.94, 1.25)	0.003
UA (μmol/L)	275.95 (217.57, 350.05)	264 (217.10, 334.60)	351.1 (267.20, 396.75)	0.004
AST (U/L)	21.00 (18.00, 26.00)	22.00 (19.00, 27.00)	20.00 (17.00, 22.50)	0.031
Follow-up information				
Age at last follow-up (years)	15.23 (13.24, 17.78)	14.94 (12.95, 17.47)	17.03 (16.04, 18.69)	< 0.001
Follow-up duration (years)	5.41 (3.96, 6.78)	5.18 (3.94, 6.62)	5.93 (4.72, 7.55)	0.086
Medication regimen, *n *(%)				0.119
Symptomatic treatment	4 (1.79)	3 (1.52)	1 (3.70)	
PDN	3 (1.34)	2 (1.02)	1 (3.70)	
PDN + IS	49 (21.88)	47 (23.86)	2 (7.41)	
RASB	19 (8.48)	17 (8.63)	2 (7.41)	
RASB + PDN	4 (1.79)	3 (1.52)	1 (3.70)	
RASB + PDN + IS	145 (64.73)	125 (63.45)	20 (74.07)	
Tonsillectomy performed, *n *(%)	10 (4.46)	7 (3.55)	3 (11.11)	0.198
Gross hematuria status, *n *(%)				0.054
RGH	74 (33.04)	67 (34.01)	7 (25.93)	
IGH	86 (38.39)	79 (40.10)	7 (25.93)	
NGH	64 (28.57)	51 (25.89)	13 (48.15)	
Proteinuria remission status, *n *(%)				< 0.001
CRP	153 (68.30)	136 (69.04)	17 (62.96)	
PRP	65 (29.02)	59 (29.95)	6 (22.22)	
NRP	6 (2.68)	2 (1.02)	4 (14.81)	
TA-H (/μL)	46.53 (19.00, 102.35)	49.21 (20.07, 104.66)	30.02 (17.91, 92.51)	0.257
TA-UPCR (μg/mg)	96.44 (34.57, 243.35)	95.27 (34.99, 238.74)	132.30 (38.01, 356.29)	0.268
eGFR at last follow-up (ml/min/1.73 m^2^)	109.05 (100.80, 113.00)	109.8 (105.00, 113.70)	82.6 (72.85, 87.95)	-
eGFR decline ≥ 30%, *n* (%)	5 (2.23)	0 (0.00)	5 (18.52)	-
eGFR decline ≥ 50%, *n* (%)	3 (1.34)	0 (0.00)	3 (11.11)	-
Kidney failure, *n*(%)	3 (1.34)	0 (0.00)	3 (11.11)	-
Kidney remission, *n*(%)				0.255
Complete remission without relapse	63 (28.12)	55 (27.92)	8 (29.63)	
Relapse after complete remission	96 (42.86)	88 (44.67)	8 (29.63)	
No remission/kidney failure	65 (29.02)	54 (27.41)	11 (40.74)	

Continuous variables are expressed as mean ± SD or median (IQR), and categorical variables as number (%). Based on whether poor prognosis occurred, patients were divided into non-endpoint group and endpoint group. *BMI* body mass index, *SBP* systolic blood pressure, *DBP* diastolic blood pressure, *MAP* mean arterial pressure, *M* mesangial hypercellularity, *E* endocapillary hypercellularity, *S* segmental glomerulosclerosis, *T* tubular atrophy/interstitial fibrosis, *C* cellular/fibrocellular crescents, *UACR* urinary albumin-to-creatinine ratio, *UPCR* urinary protein-to-creatinine ratio, *24 h-Ualb* 24-h urinary albumin excretion, *24 h-UTP* 24-h urinary total protein excretion, *eGFR* estimated glomerular filtration rate, *Scr* serum creatinine, *CysC* cystatin C, *UA* uric acid, *AST* aspartate aminotransferase, *PDN* oral prednisolone, *IS* other immunosuppressants, *RASB* renin-angiotensin system blockers, *RGH* recurrent gross hematuria, *IGH* isolated gross hematuria, *NGH* no gross hematuria, *CRP* complete remission of proteinuria, *PRP* partial remission of proteinuria, *NRP* no remission of proteinuria, *TA-H* time-averaged urine red blood cell count, *TA-UPCR* time-averaged urinary protein-to-creatinine ratio

### Poor prognosis

#### Differences between endpoint and non-endpoint groups

Based on whether poor progenosis (sustained eGFR < 90 mL/min/1.73 m^2^ for ≥ 3 months) occurred, patients were divided into non-endpoint and endpoint groups. Significant intergroup differences (*p*-value < 0.05) were observed in sex, birth weight, gestational age, family history of kidney disease, age at onset and biopsy, Oxford classification E and S lesions, systolic blood pressure (SBP), diastolic blood pressure (DBP), mean arterial pressure (MAP), height, weight, body mass index (BMI), eGFR at biopsy, Scr, cystatin C (CysC), uric acid (UA), aspartate aminotransferase (AST), age at last follow-up, and proteinuria remission status during follow-up.

#### Independent predictive factors for poor prognosis

**Fig. 1:**
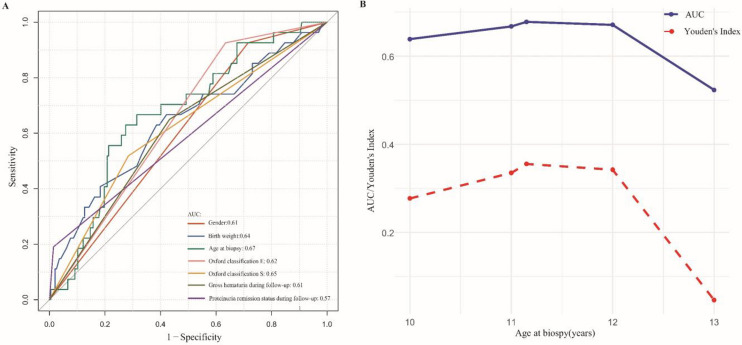
ROC analysis of predictive factors and age thresholds at biopsy for poor prognosis. **A** ROC curve analysis of independent associated factors for poor progenies. **B** Sensitivity analysis of age thresholds at biopsy based on ROC curves. *ROC*, receiver operating characteristic; *AUC*, area under the curve

Multivariate logistic regression (Table 2) identified male sex, older age at biopsy, Oxford classification E1 and S1 lesions, NGH, and NRP during follow-up as independent risk factors for poor prognosis, while higher birth weight was protective. The AUC for each predictor is shown in Fig. 1A. The optimal cutoff for birth weight was 3.21 kg (Youden index = 0.25), and for age at biopsy was 11.15 years (Youden index = 0.36). To enhance the clinical applicability, sensitivity analyses were conducted using age cutoffs of 10, 11, 12, and 13 years. The 12-year threshold showed comparable performance to the optimal cutoff and outperformed other tested values (Fig. 1B).

**Table 2:** Logistic regression analysis of independent predictive factors for poor prognosis

Variables	Univariate analysis			Multivariate analysis		
	*β*	*p-value*	Univariate OR (95% CI)	*β*	*p-value*	Univariate OR (95% CI)
Sex						
Female			1.00 (Reference)			1.00 (Reference)
Male	1.60	0.033	4.96 (1.14 ~ 21.66)	2.23	0.011	9.30 (1.68 ~ 51.56)
Gestational age						
Preterm birth			1.00 (Reference)			
Term birth	−1.80	0.024	0.17 (0.03 ~ 0.79)			
Birth weight (kg)	−1.12	0.015	0.32 (0.13 ~ 0.80)	−1.35	0.020	0.26 (0.08 ~ 0.81)
Family history of kidney disease						
No			1.00 (Reference)			
Yes	1.33	0.004	3.78 (1.52 ~ 9.40)			
Age at onset (years)	0.18	0.023	1.20 (1.03 ~ 1.41)			
Age at biopsy (years)	0.21	0.006	1.23 (1.06 ~ 1.44)	0.20	0.038	1.22 (1.01 ~ 1.48)
Oxford classification M score						
0			1.00 (Reference)			
1	0.21	0.639	1.23 (0.52 ~ 2.89)			
Oxford classification E score						
0			1.00 (Reference)			1.00 (Reference)
1	1.00	0.017	2.71 (1.20 ~ 6.13)	1.27	0.015	3.56 (1.28 ~ 9.86)
Oxford classification S score						
0			1.00 (Reference)			1.00 (Reference)
1	1.97	0.008	7.20 (1.66 ~ 31.29)	2.19	0.008	8.91 (1.77 ~ 44.83)
Oxford classification T score						
0			1.00 (Reference)			
1	−0.00	0.992	1.00 (0.41 ~ 2.43)			
2	0.77	0.358	2.17 (0.42 ~ 11.30)			
Oxford classification C score						
0			1.00 (Reference)			
1	0.04	0.921	1.05 (0.44 ~ 2.51)			
2	0.35	0.585	1.42 (0.41 ~ 4.96)			
Height (cm)	0.05	0.002	1.05 (1.02 ~ 1.08)			
Weight (kg)	0.05	< 0.001	1.05 (1.02 ~ 1.08)			
BMI (kg/m^2^)	0.14	0.005	1.16 (1.05 ~ 1.28)			
SBP (mmHg)	0.05	0.002	1.05 (1.02 ~ 1.08)			
DBP (mmHg)	0.04	0.042	1.04 (1.01 ~ 1.08)			
MAP (mmHg)	0.05	0.008	1.05 (1.01 ~ 1.10)			
eGFR at biopsy (ml/min/1.73 m^2^)	−0.01	0.101	0.99 (0.97 ~ 1.00)			
Scr (mmol/L)	0.02	0.004	1.03 (1.01 ~ 1.04)			
CysC (mg/L)	1.89	0.004	6.63 (1.82 ~ 24.17)			
UA (μmol/L)	0.01	0.005	1.01 (1.01 ~ 1.01)			
Gross hematuria status during follow-up						
RGH			1.00 (Reference)			1.00 (Reference)
IGH	−0.16	0.768	0.85 (0.28 ~ 2.54)	−0.30	0.636	0.74 (0.21 ~ 2.59)
NGH	0.89	0.077	2.44 (0.91 ~ 6.56)	1.25	0.048	3.48 (1.01 ~ 11.96)
Proteinuria remission status during follow-up						
CRP			1.00 (Reference)			1.00 (Reference)
PRP	−0.21	0.680	0.81 (0.31 ~ 2.17)	−1.01	0.090	0.36 (0.11 ~ 1.17)
NRP	2.77	0.002	16.00 (2.72 ~ 94.00)	2.97	0.017	19.46 (1.72 ~ 220.34)
TA-UPCR (μg/mg)	0.01	0.010	1.01 (1.01 ~ 1.01)			
TA-H (/μL)	−0.00	0.420	1.00 (1.00 ~ 1.00)			

*OR* odds ratio, *CI* confidence interval, *M* mesangial hypercellularity, *E* endocapillary hypercellularity, *S* segmental glomerulosclerosis, *T* tubular atrophy/interstitial fibrosis, *C* cellular/fibrocellular crescents, *BMI* body mass index, *SBP* systolic blood pressure, *DBP* diastolic blood pressure, *MAP* mean arterial pressure, *eGFR* estimated glomerular filtration rate, *Scr* serum creatinine, *CysC* cystatin C, *UA* uric acid, *RGH* recurrent gross hematuria, *IGH* isolated gross hematuria, *NGH* no gross hematuria, *CRP* complete remission of proteinuria, *PRP* partial remission of proteinuria, *NRP* no remission of proteinuria, *TA-UPCR* time-averaged urinary protein-to-creatinine ratio, *TA-H* time-averaged hematuria

#### Time-dependent predictors of eGFR slope

**Fig. 2:**
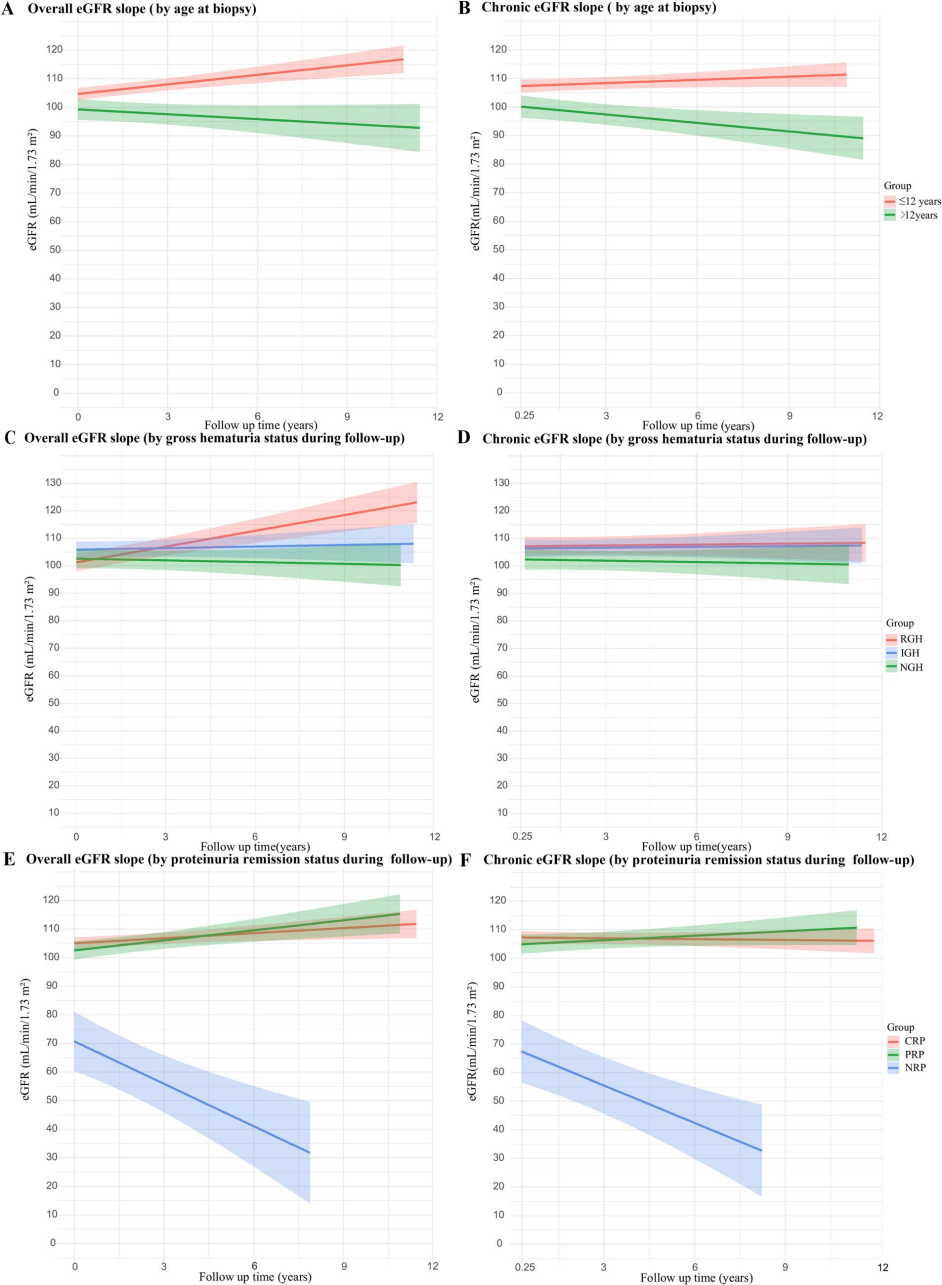
Effects of time-dependent predictors on eGFR slope. **A/B**, eGFR slopes grouped by age at biopsy; **C/D**, eGFR slopes grouped by gross hematuria status during follow-up; **E/F**, eGFR slopes grouped by proteinuria remission status during follow-up. *eGFR*, estimated glomerular filtration rate; *RGH*, recurrent gross hematuria; *IGH*, isolated gross hematuria; *NGH*, no gross hematuria; *CRP*, complete remission of proteinuria; *PRP*, partial remission of proteinuria; *NRP*, no remission of proteinuria

LMM analysis revealed that age at biopsy, gross hematuria status, and proteinuria remission status during follow-up exerted significant time-dependent effects on eGFR slope, while birth weight and Oxford classification S and E lesions did not show significant associations (Supplementary Table 2). For overall eGFR, the slope differed significantly between patients aged < 12 years and those ≥ 12 years at biopsy, as well as between patients with RGH and those with IGH/NGH (all *p-values*< 0.05). The CRP group showed a significantly slower decline than the NRP group (*p*-value < 0.05), but not the PRP group (*p*-value > 0.05). For chronic eGFR, similar trends were observed for age at biopsy and proteinuria remission status during follow-up, while differences among gross hematuria groups were not significant (*p*-value > 0.05) (Table 3, Fig. 2).

**Table 3:**
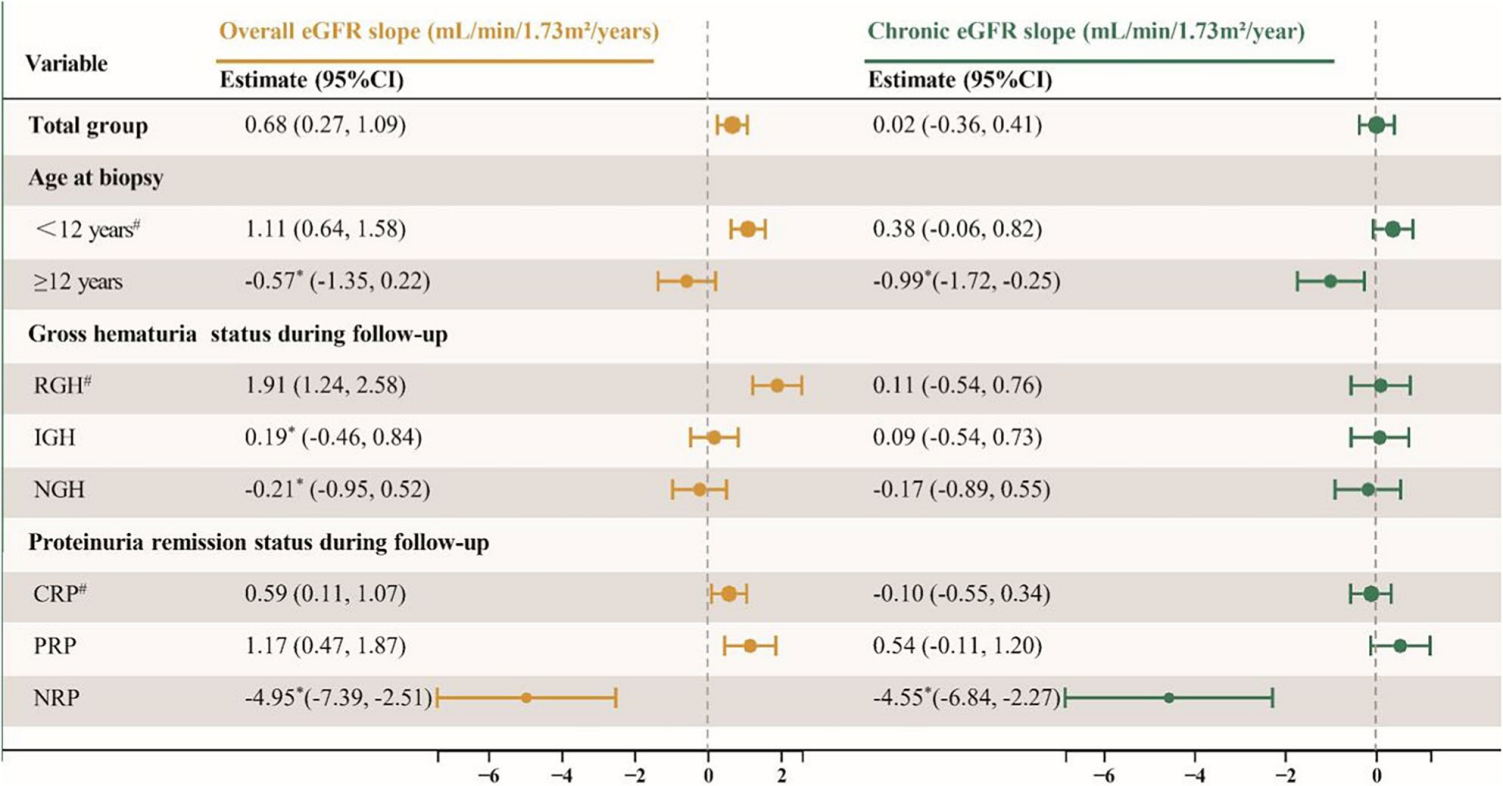
Differences in eGFR slope among significant interaction variables

^#^Reference group; * indicates *p-value* < 0.05 compared with the reference group. *eGFR* estimated glomerular filtration rate, *CI* confidence interval, *RGH* recurrent gross hematuria, *IGH* isolated gross hematuria, *NGH* no gross hematuria, *CRP* complete remission of proteinuria, *PRP* partial remission of proteinuria, *NRP* no remission of proteinuria

### Complete kidney remission

#### Dynamic patterns of complete kidney remission and relapse

**Fig. 3:**
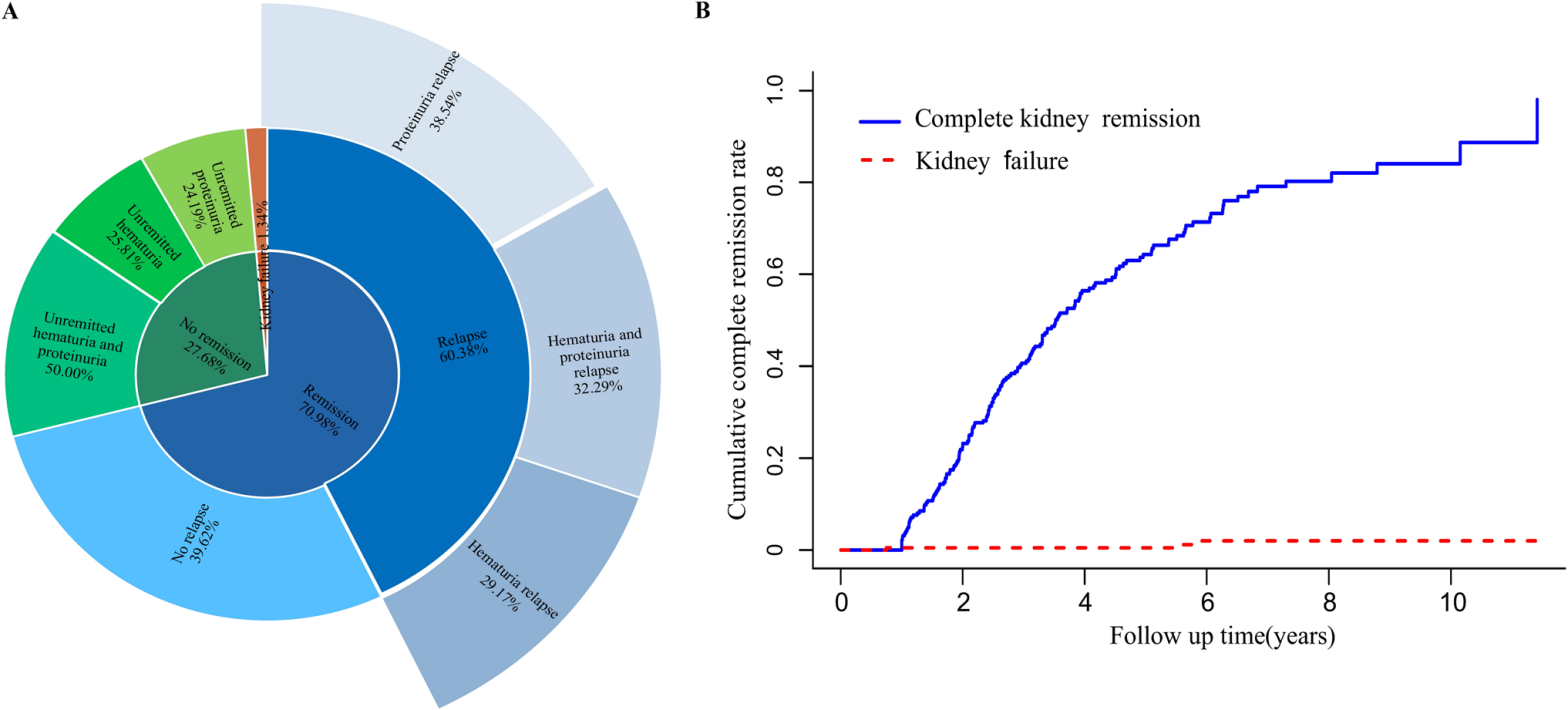
Complete kidney remission in pediatric patients with IgA nephropathy. **A** Distribution of kidney remission and relapse status. **B** Cumulative incidence curve of complete kidney remission

By the last follow-up, 70.98% achieved complete kidney remission, with a median time of 3.52 years (95% CI: 3.14–3.95). The cumulative incidence of complete kidney remission at 2, 5, and 10 years post-biopsy was 23.21% (95% CI: 17.67–28.76%), 64.33% (95% CI: 57.58–71.08%), and 84.03% (95% CI: 76.64–91.43%), respectively. Among those in remission, 39.62% (63/159) remained relapse-free, while 60.38% (96/159) relapsed, including hematuria (29.17%), proteinuria (38.54%), or both (32.29%) (Fig. 3).

#### Independent predictive factors for complete kidney remission

Fine–Gray competing risk analysis (Table 4) identified antecedent infection, kidney IgM deposition of 2 +, elevated serum IgM, increased pathological casts count, and PDN treatment as independent protective factors for complete kidney remission, whereas prolonged disease duration before biopsy and elevated tubular casts count were independent risk factors.

**Table 4:** Fine–Gray competing risks model analysis for complete kidney remission

Variables	Univariate analysis		Multivariate analysis	
	*p-value*	Univariate OR (95% CI)	*p-value*	Univariate OR (95% CI)
Family history of kidney disease				
No		1.00 (Reference)		
Yes	0.011	0.49 (0.28 ~ 0.85)		
Age at biopsy (years)	0.003	0.91 (0.86 ~ 0.97)		
Disease duration before biopsy (months)	0.003	0.99 (0.98 ~ 1.00)	0.016	0.98 (0.97 ~ 1.00)
Antecedent infection				
No		1.00 (Reference)		1.00 (Reference)
Yes	0.037	1.39 (1.02 ~ 1.89)		1.80 (1.16 ~ 2.79)
Oxford classification T score				
0		1.00 (Reference)		
1	0.025	0.68 (0.49 ~ 0.95)		
2	0.240	0.55 (0.20 ~ 1.50)		
Degree of kidney IgA deposition				
1 +		1.00 (Reference)		
2 +	0.050	0.37 (0.13 ~ 1.00)		
3 +	0.210	0.58 (0.25 ~ 1.35)		
4 +	0.021	0.45 (0.23 ~ 0.89)		
Degree of kidney IgM deposition				
-		1.00 (Reference)		1.00 (Reference)
1 +	0.320	1.24 (0.81 ~ 1.88)	0.980	1.01 (0.60 ~ 1.69)
2 +	0.002	1.90 (1.26 ~ 2.87)	0.043	1.77 (1.02 ~ 3.08)
3 +	0.310	1.40 (0.73 ~ 2.69)	0.260	1.79 (0.65 ~ 4.90)
Degree of kidney C3 deposition				
-		1.00 (Reference)		
1 +	0.910	1.05 (0.44 ~ 2.53)		
2 +	0.480	0.85 (0.54 ~ 1.33)		
3 +	0.047	0.65 (0.42 ~ 0.99)		
4 +	0.330	0.81 (0.53 ~ 1.24)		
Proteinuria status at biopsy				
No proteinuria		1.00 (Reference)		
Non-nephrotic syndrome level	0.007	0.59 (0.40 ~ 0.86)		
Nephrotic syndrome level	0.048	0.63 (0.40 ~ 1.00)		
Height (cm)	0.049	0.99 (0.98 ~ 1.00)		
SBP (mmHg)	< 0.001	0.98 (0.96 ~ 0.99)		
MAP (mmHg)	0.005	0.97 (0.96 ~ 0.99)		
Urinary WBCs (/μL)	0.007	1.00 (1.00 ~ 1.00)		
Tubular casts (/μL)	0.048	1.02 (1.00 ~ 1.05)	0.038	0.90 (0.81 ~ 0.99)
Pathological casts (/μL)	< 0.001	1.05 (1.03 ~ 1.07)	0.031	1.24 (1.05 ~ 1.47)
MONO (10^9^/L)	0.006	1.65 (1.15 ~ 2.35)		
PT (S)	< 0.001	1.02 (1.01 ~ 1.03)		
ALP (U/L)	0.043	1.00 (1.00 ~ 1.00)		
Ca^2+^ (mmol/L)	0.026	0.36 (0.15 ~ 0.88)		
IgM (g/L)	< 0.001	1.38 (1.16 ~ 1.64)	< 0.001	1.50 (1.21 ~ 1.86)
Medication regimen				
Symptomatic treatment		1.00 (Reference)		1.00 (Reference)
PDN	0.077	2.24 (0.91 ~ 5.49)	0.016	9.03 (1.51 ~ 53.88)
PDN + IS	0.048	0.49 (0.25 ~ 0.99)	0.600	0.69 (0.17 ~ 2.78)
RASB	0.022	0.40 (0.18 ~ 0.88)	0.290	0.49 (0.13 ~ 1.84)
RASB + PDN	0.550	0.81 (0.40 ~ 1.64)	0.740	1.31 (0.28 ~ 6.14)
RASB + PDN + IS	< 0.001	0.29 (0.15 ~ 0.57)	0.260	0.45 (0.11 ~ 1.78)
Tonsillectomy performed				
No		1.00 (Reference)		
Yes	0.032	0.37 (0.15 ~ 0.92)		

*OR* odds ratio, *CI* confidence interval, *T* tubular atrophy/interstitial fibrosis, *IgG* immunoglobulin G, *IgM* immunoglobulin M, *C3* complement component 3, *SBP* systolic blood pressure, *MAP* mean arterial pressure *WBCs* white blood cells, *MONO* monocyte count, *PT* prothrombin time, *ALP* alkaline phosphatase, *Ca*^*2*^*⁺* serum calcium concentration, *PDN* oral prednisolone, *IS* immunosuppressants, *RASB* renin–angiotensin system blocker

#### Relationship between complete kidney remission and poor prognosis

Group comparisons and logistic regression analysis showed no significant associations between kidney remission and poor prognosis (*p*-value > 0.05) (Tables 1 and 5), suggesting that achieving complete kidney remission did not directly predict endpoint events in this cohort.

**Table 5:** Logistic regression analysis of the association between kidney remission and poor prognosis

Variables	Univariate analysis			Multivariate analysis^1^				
	*β*	*p-value*	Univariate OR (95% CI)	*β*	*p-value*		Univariate OR (95% CI)	
Kidney remission								
Complete remission without relapse			1.00 (Reference)					1.00 (Reference)
Relapse after complete remission	−0.47	0.374	0.63 (0.22 ~ 1.76)	−0.48		0.437		0.62 (0.19 ~ 2.06)
No remission/kidney failure	0.34	0.503	1.40 (0.52 ~ 3.75)	−0.29		0.659		0.75 (0.21 ~ 2.68)

^1^Adjusted for sex, birth weight, age at biopsy, Oxford classification E and S lesions, and gross hematuria status during follow-up. *OR* odds ratio, *CI* confidence interval

## Discussion

IgAN is one of the major causes of chronic kidney disease (CKD) and kidney failure among pediatric glomerulopathies, with marked heterogeneity in clinical course and prognosis. In this study, we systematically analyzed baseline characteristics, clinicopathological features at biopsy, and follow-up data from 224 pediatric patients with IgAN. We identified independent predictors of poor prognosis and evaluated their impact on eGFR slope.

In addition, we analyzed the dynamic patterns of complete kidney remission and relapse, identified predictive factors associated with remission, and examined the influence of remission on poor prognosis. This integrative analysis offers theoretical evidence for early risk stratification and targeted intervention in pediatric IgAN.

### Poor prognosis

Consistent with previous studies, the pediatric IgAN cohort in this study showed an overall indolent course. Over a median follow-up of 5.41 years, only 2.23% experienced ≥ 30% eGFR decline, and kidney failure incidence was 1.34%. These low event rates highlight the limitations of relying solely on traditional hard endpoints for prognosis in pediatric IgAN, given the long natural history and the difficulty of capturing a full life-course through clinical follow-up. To enhance prognostic sensitivity, we pre-specified a dual-indicator approach. First, in light of children's higher kidney reserve and longer life expectancy, we defined sustained eGFR decline to < 90 mL/min/1.73 m^2^ for ≥ 3 months as a pediatric surrogate endpoint, enabling earlier detection of kidney impairment and broader therapeutic windows [17]. Using this criterion, 12.05% met this endpoint, underscoring the need for early warning systems. Second, to overcome the limitations of single time-point measurements of eGFR and to better capture the chronic disease trajectory, we assessed longitudinal outcomes using the eGFR slope. To minimize the confounding effects of acute-phase treatments, both the overall eGFR slope (entire disease course) and the chronic eGFR slope (excluding the first three months) were calculated [18–20]. Prior studies indicate acute effects wane over time, with follow-ups beyond three years validating overall eGFR slope as a reliable long-term prognostic marker.

Male sex emerged as an independent risk factor for poor prognosis. Previous studies have reported that male patients often exhibited more severe pathology, higher proteinuria, and faster kidney function decline [21, 22]. Potential mechanisms include sex hormone–mediated differences: estrogen exerts anti-fibrotic and anti-apoptotic effects, whereas testosterone tends to promote inflammation, fibrosis, and apoptosis. Animal studies have demonstrated that female mice maintain higher nitric oxide (NO) levels and basal nitric oxide synthase (NOS) activity, with lower pro-inflammatory mediators and reduced kidney oxidative stress. Furthermore, differences in biopsy indications, follow-up duration, and endpoint definitions may also contribute to sex-related prognostic disparities [21].

Younger age at biopsy was associated with more favorable outcomes. In younger children, eGFR tended to rise, likely reflecting compensatory kidney growth, while older children exhibited dynamics resembling adult-type decline. This aligns with findings from the Validation Study of the Oxford Classification of IgAN (VALIGA) cohort, which showed that progression risk increased with age and plateaued around 23 years, supporting the hypothesis that kidney regenerative capacity diminishes with increasing age [23]. Similarly, the International IgA Nephropathy Network (IIGANN) also observed a nonlinear eGFR trajectory, with increases prior to adolescence followed by progressive decline, and smaller early gains predicting faster deterioration [12, 13]. Our findings quantitatively characterized eGFR slope in different age groups at biopsy, identifying age 12 as a potential transition point from compensatory growth to pathological deterioration.

Higher birth weight was identified as a protective factor, suggesting that low birth weight (LBW) or small-for-gestational-age (SGA)—the proxy for intrauterine growth restriction—may predispose children with IgAN to kidney dysfunction. Prior studies have linked LBW or SGA status to increased risks of hypertension, proteinuria, and CKD progression in adulthood [24, 25]. In a Japanese pediatric IgAN cohort (*n* = 50, mean age at biopsy: 11.2 years, median follow-up duration: 8.8 years), LBW was associated with hypertension, glomerulosclerosis, and CKD [26]. Similarly, a Norwegian study (*n* = 471, mean age at biopsy: 23.8 years, median follow-up duration: 6.7 years) reported that LBW and/or SGA were significantly associated with higher kidney failure risk, particularly in males [27]. Pathologically, affected patients had larger glomerular areas, suggesting compensatory hypertrophy due to reduced nephron number [28]. LBW and/or SGA may act as a “first hit” in IgAN by reducing nephron endowment, leading to compensatory glomerular hypertrophy and increased filtration burden, while postnatal stressors such as hypertension and proteinuria constitute “second hits” that accelerate disease progression [28].

Pathological findings from the Oxford classification, particularly E1 and S1 lesions, were independently associated with poor prognosis. In children, S1 lesions often represent active podocyte injury (e.g., tip lesions or podocyte hypertrophy), correlating with heavier proteinuria and faster eGFR decline, and may respond to immunosuppressive therapy. Conversely, S1 lesions in adults more commonly reflect chronic scarring [29, 30]. At present, S1 lesions have been widely recognized as strong predictors of poor prognosis in pediatric IgAN. A large Chinese pediatric study (*n* = 1243, mean age at biopsy: 13.7 years, median follow-up: 7.2 years) and the European VALIGA study (*n* = 1147, 15% pediatric) both confirmed the association of S1 lesions with adverse outcomes [31, 32]. In contrast, the prognostic significance of E1 lesions remains controversial [33–35]. A meta-analysis showed that E1 lesions were significantly associated with an increased risk of poor prognosis in non-Asian pediatric patients with IgAN, whereas this association was not statistically significant in Asian patients [36]. A Swedish cohort (*n* = 99, mean age at biopsy: 12 years, mean follow-up: 13 years) reported that E1 lesions predicted poor prognosis only when combined with proteinuria [37]. These inconsistencies may stem from lesion reversibility, ethnic differences, biopsy timing, baseline severity, endpoint definitions, inter-observer variability in lesion scoring, and limited statistical power due to low event rates [38]. In our cohort, E1 and S1 lesions were independently associated with the binary poor-prognosis endpoint, yet neither showed a significant effect on eGFR slope over time in the LMMs (overall or chronic). This apparent divergence likely reflects the distinction between a threshold-based endpoint, which can be triggered by active and partially reversible lesions (E1/S1), and the longitudinal slope, which primarily reflects chronic decline driven by persistent proteinuria control and age-related trajectories. Additionally, the relatively low event rate and inter-observer variability in pediatric lesion scoring may have limited the power to detect modest time-dependent effects of E1/S1 lesions on eGFR slope.

Gross hematuria, commonly triggered by mucosal infections, is a frequent manifestation in children with IgAN [6, 39]. While adult studies have linked gross hematuria to differing long-term outcomes, evidence in children remains limited [40–42]. Our study showed that patients with NGH had a higher risk of poor prognosis than those with RGH. eGFR slope analyses supported this, with RGH associated with more favorable trajectories. Prior studies suggest the chemokine (C-X3-C motif) receptor 1–fractalkine (CX3CR1–FKN) axis may mediate both hematuria and kidney protection by facilitating immune cell infiltration during flares, while also suppressing fibrosis [43, 44]. Additional endogenous mechanisms, including the Heme oxygenase-1 (HO-1) pathway, haptoglobin–CD163 axis, iron chelators, and antioxidant defenses, may protect against hematuria-induced injury [45]. Clinically, patients with RGH typically receive prompt treatment, while those with NGH may experience insidious disease progression and delayed treatment, contributing to worse kidney outcomes [46, 47].

TA-UPCR is a well-established prognostic marker in IgAN and has been incorporated into surrogate endpoints for disease progression to kidney failure or ≥ 30% eGFR decline [48]. Pathophysiologically, persistent proteinuria accelerates kidney damage by including tubular chemokine expression, complement activation, and inflammatory cell infiltration, ultimately driving tubulointerstitial fibrosis [49]. In our study, CRP was linked to more favorable overall and chronic eGFR slopes. The 2024 draft KDIGO guideline refines proteinuria targets: for adults, 24 h-UTP < 500 mg/day is recommended, ideally < 300 mg/day; for children, stricter criteria are proposed, with 24 h-UTP ≤ 200 mg/day (or ≤ 400 mg/1.73 m^2^/day), or UPCR ≤ 200 mg/g [50]. These findings underscore the importance of early and sustained proteinuria control in preserving long-term kidney function.

### Complete kidney remission

Current studies on kidney remission in IgAN remain limited, and a standardized definition is lacking. In 2012, Shima et al. defined remission as the absence of hematuria and proteinuria, but without specifying duration. Among 96 untreated children with mild IgAN, 59.4% achieved spontaneous remission, with 5- and 10-year cumulative rates of 57.5% and 77.4%, respectively; however, 17.5% relapsed, with cumulative relapse rates of 79.9% and 67.9% [7]. In 2013, the Japanese Society of Nephrology (JSN) proposed a definition requiring three consecutive negative urinalyses over six months [51]. Using this definition, Matsushita et al. reported a 50.9% remission rate in a cohort of Japanese children (*n* = 53, median onset age: 10 years, median follow-up: 9.9 years) and identified UPCR < 500 mg/g at 2 years post-onset as an independent predictor [52]. In 2024, Antonucci et al. retrospectively analyzed Italian children with IgAN (*n* = 153, mean onset age: 10.6 years, median follow-up: 5.8 years) and proposed a stricter “clinical complete remission”: drug withdrawal ≥ 2 years, absence of hematuria/proteinuria, normal BP, and eGFR ≥ 90 mL/min/1.73 m^2^, under which the 10-year remission rate was 43%, though relapses persisted. Further analysis identified younger age at onset and S0 lesions as protective factors [53]. With an even stricter criterion (drug withdrawal ≥ 5 years, follow-up ≥ 10 years), 42% achieved remission, but two relapsed. Based on the 2024 IPNA Practice Recommendations, we defined complete kidney remission as sustained absence of hematuria and proteinuria for ≥ 1 year with eGFR ≥ 90 mL/min/1.73 m^2^ [14]. Under this definition, 70.98% achieved remission, with cumulative rates increasing over time. However, 60.38% subsequently relapsed, mainly due to recurrent proteinuria. Further analysis identified antecedent infection, kidney IgM deposition of 2 +, elevated serum IgM, elevated pathological casts count, and PDN treatment as protective factors for remission, while prolonged disease duration before biopsy and elevated tubular casts count were risk factors for remission. Neither group comparisons nor logistic regression showed significant association between kidney remission and poor prognosis, possibly due to complex relapse dynamics, residual confounding, or limited sample size. These findings suggest that current remission criteria—based on proteinuria and hematuria resolution and stable eGFR—may underestimate residual disease activity and relapse potential. Remission in IgAN is not necessarily durable; relapse can occur despite strict clinical remission. Future research should refine kidney remission definitions and establish relapse risk models incorporating histopathology, biomarkers, and long-term data to improve prognostication in pediatric IgAN.

### Limitations

This study has several limitations. First, as a single-center retrospective study, it is subject to selection bias and regional specificity. Second, potential confounders such as genetics, lifestyle, and comorbidities were not fully controlled. Third, the median follow-up duration was relatively short for capturing long-term outcomes. Fourth, the lack of a standardized definition for complete kidney remission hampers cross-study comparability. Fifth, although treatment data were included, the evaluation of therapeutic strategies and their prognostic effects were insufficient.

In conclusion, this study systematically evaluated prognostic determinants and kidney remission characteristics in pediatric IgAN, highlighting the role of early intervention and individualized risk assessment. Future research should leverage multicenter cohorts and integrate multi-omics approaches—including genomic, metabolic, and inflammatory profiling—to enhance prognostic accuracy and guide precision therapy in pediatric IgAN.

## Supplementary Information

Below is the link to the electronic supplementary material.

Graphical abstract (PPTX 1824 KB)

Supplementary file2 (DOCX 290 KB)

## Data Availability

The data concerning the findings of this study are available from the corresponding authors upon reasonable request.
