# Supplementary material for: Prognosis and predictive factors in pediatric IgA nephropathy
Source: Pediatr Nephrol. 2025 Nov 13;41(3):731–45. doi: 10.1007/s00467-025-06988-8 (PMC12852140; doi:10.1007/s00467-025-06988-8)
Supplement: Supplementary file 1 — Graphical abstract (PPTX 1824 KB) [file 467_2025_6988_MOESM1_ESM.pptx]

## Slide 1
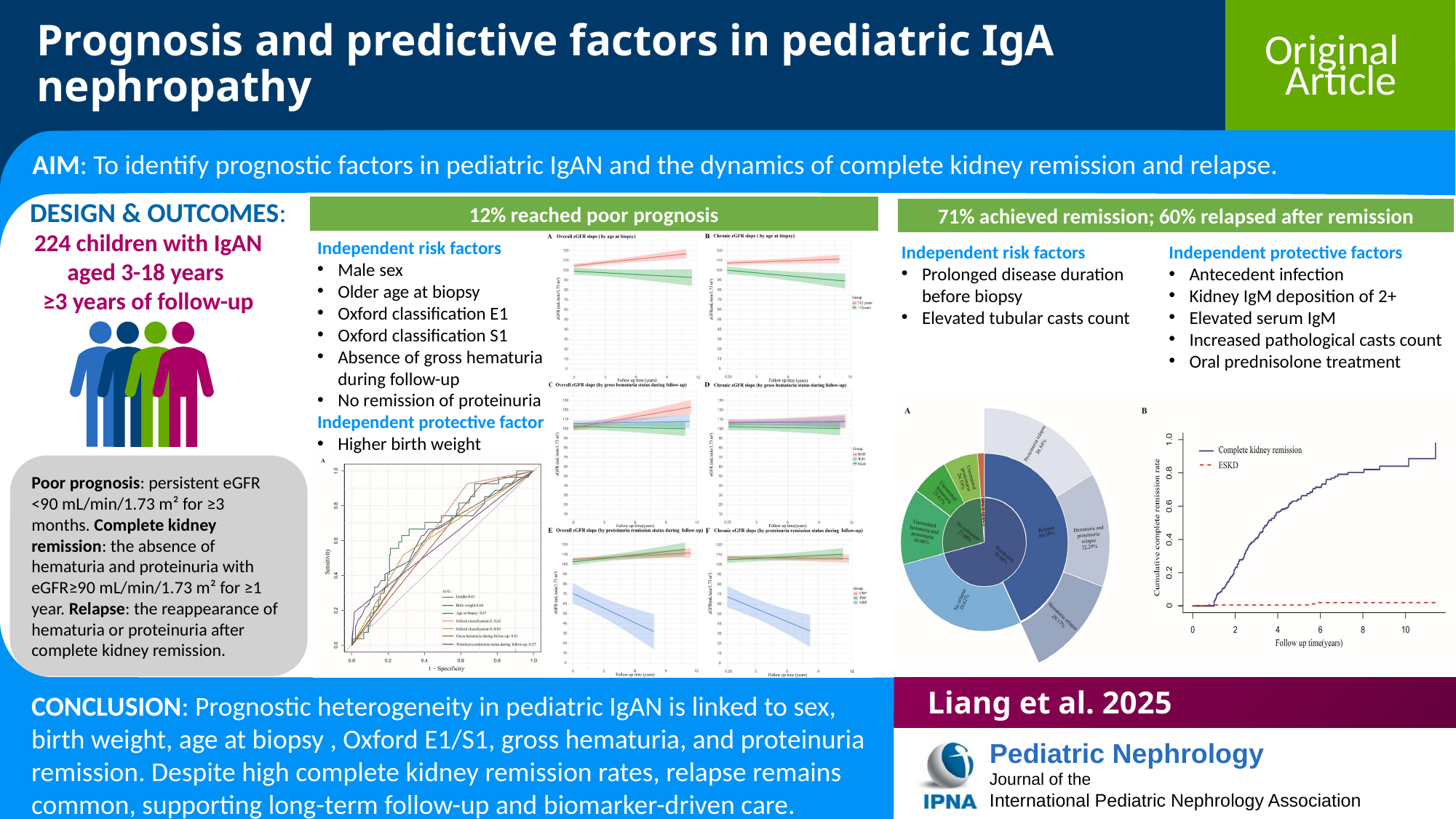

Prognosis and predictive factors in pediatric IgA nephropathy
AIM: To identify prognostic factors in pediatric IgAN and the dynamics of complete kidney remission and relapse.
DESIGN & OUTCOMES:
12% reached poor prognosis
71% achieved remission; 60% relapsed after remission
224 children with IgAN
aged 3-18 years
≥3 years of follow-up
Independent risk factors
Male sex
Older age at biopsy
Oxford classification E1
Oxford classification S1
Absence of gross hematuria during follow-up
No remission of proteinuria
Independent protective factor
Higher birth weight
Independent risk factors
Prolonged disease duration before biopsy
Elevated tubular casts count
Independent protective factors
Antecedent infection
Kidney IgM deposition of 2+
Elevated serum IgM
Increased pathological casts count
Oral prednisolone treatment
Poor prognosis: persistent eGFR <90 mL/min/1.73 m² for ≥3 months. Complete kidney remission: the absence of hematuria and proteinuria with eGFR≥90 mL/min/1.73 m² for ≥1 year. Relapse: the reappearance of hematuria or proteinuria after complete kidney remission.
Liang et al. 2025
CONCLUSION: Prognostic heterogeneity in pediatric IgAN is linked to sex, birth weight, age at biopsy , Oxford E1/S1, gross hematuria, and proteinuria remission. Despite high complete kidney remission rates, relapse remains common, supporting long-term follow-up and biomarker-driven care.
