# Supplementary material for: Prognosis and predictive factors in pediatric IgA nephropathy
Source: Pediatr Nephrol. 2025 Nov 13;41(3):731–45. doi: 10.1007/s00467-025-06988-8 (PMC12852140; doi:10.1007/s00467-025-06988-8)
Supplement: Supplementary file 2 — Supplementary file2 (DOCX 290 KB) [file 467_2025_6988_MOESM2_ESM.docx]

**Supplementary material**

**The prognosis and predictive factors in pediatric IgA nephropathy**

**Pediatric Nephrology**

Wenpei Liang^1^, Yonghua He^1^, Xueqing Ma^1^, Panpan Shao^1^, Ling Guo^1^, Jianhua Zhou^1^, Yu Zhang^1^, Huiqing Yuan^1^, Liru Qiu^1,2,3^

^1^ Department of Pediatrics, Tongji Hospital, Tongji Medical College, Huazhong University of Science and Technology, Wuhan, China

^2^Hubei Provincial Clinical Research Center for Child Growth, Development, and Metabolic Diseases ^3^Hubei Key Laboratory for Pediatric Genetic Metabolic and Endocrine Rare Disease

**Correspondence to:** Liru Qiu, MD. Department of Pediatrics, Tongji Hospital, Tongji Medical College, Huazhong University of Science and Technology, 1095 Jiefang Avenue, Qiaokou District, Wuhan 430030, China. Email: [liruqiutj@163.com](mailto:liruqiutj@163.com).**Supplementary** **Fig. 1 Flow diagram of included and excluded pediatric patients with IgA
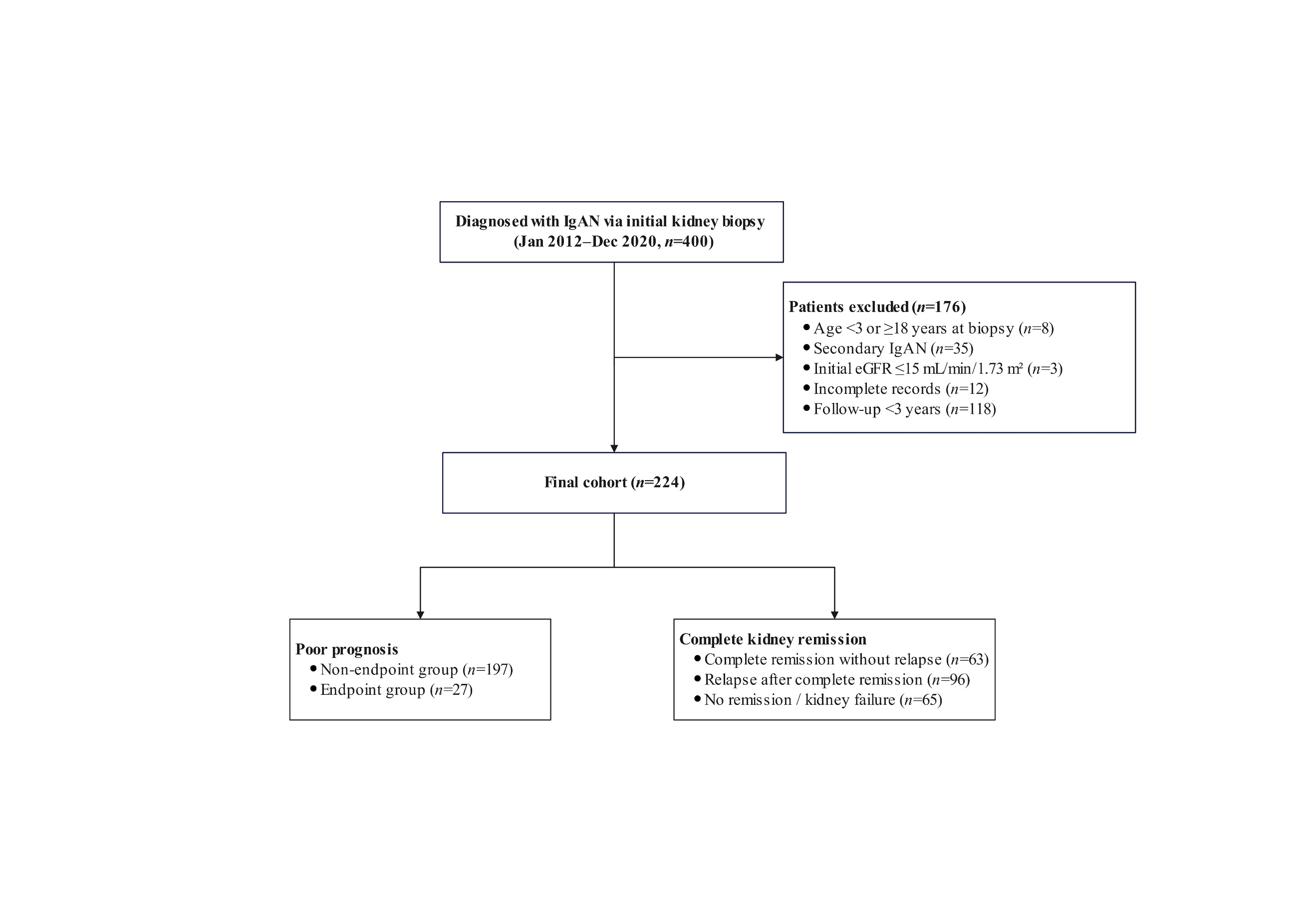
nephropathy**

*IgAN*, immunoglobulin A nephropathy; *eGFR*, estimated glomerular filtration rate.

**Supplementary Table 1 Additional clinical, pathological and** **follow-up data of pediatric patients with IgA nephropathy**

| **Variables** | **Total group**  **(n = 224)** | **Non-endpoint group**  **(n = 197)** | **Endpoint group**  **(n = 27)** | ***p-value*** |
| --- | --- | --- | --- | --- |
| **Kidney pathology** |  |  |  |  |
| Lee classification, *n*(%) |  |  |  | 0.830 |
| Grade Ⅱ | 21 (9.38) | 19 (9.64) | 2 (7.41) |  |
| Grade Ⅲ | 105 (46.88) | 94 (47.72) | 11 (40.74) |  |
| Grade Ⅳ | 82 (36.61) | 70 (35.53) | 12 (44.44) |  |
| Grade Ⅴ | 16 (7.14) | 14 (7.11) | 2 (7.41) |  |
| Types of immune complex deposition, *n*(%) |  |  |  | 0.178 |
| IgA | 116 (51.79) | 105 (53.30) | 11 (40.74) |  |
| IgA+IgG | 14 (6.25) | 14 (7.11) | 0 (0.00) |  |
| IgA+IgM | 66 (29.46) | 55 (27.92) | 11 (40.74) |  |
| IgA+IgM+IgG | 28 (12.50) | 23 (11.68) | 5 (18.52) |  |
| Presence of complement deposition, *n*(%) | 131 (58.48) | 116 (58.88) | 15 (55.56) | 0.742 |
| Degree of immune complex deposition, *n*(%) |  |  |  |  |
| IgA |  |  |  | 0.708 |
| 1+ | 14 (6.25) | 13 (6.60) | 1 (3.70) |  |
| 2+ | 14 (6.25) | 11 (5.58) | 3 (11.11) |  |
| 3+ | 26 (11.61) | 24 (12.18) | 2 (7.41) |  |
| 4+ | 170 (75.89) | 149 (75.63) | 21 (77.78) |  |
| IgM |  |  |  | 0.164 |
| - | 130 (58.04) | 119 (60.41) | 11 (40.74) |  |
| 1+ | 46 (20.54) | 38 (19.29) | 8 (29.63) |  |
| 2+ | 37 (16.52) | 30 (15.23) | 7 (25.93) |  |
| 3+ | 11 (4.91) | 10 (5.08) | 1 (3.70) |  |
| IgG |  |  |  | 0.557 |
| - | 182 (81.25) | 160 (81.22) | 22 (81.48) |  |
| 1+ | 24 (10.71) | 22 (11.17) | 2 (7.41) |  |
| 2+ | 10 (4.46) | 9 (4.57) | 1 (3.70) |  |
| 3+ | 8 (3.57) | 6 (3.05) | 2 (7.41) |  |
| C3 |  |  |  | 0.528 |
| - | 95 (42.41) | 83 (42.13) | 12 (44.44) |  |
| 1+ | 12 (5.36) | 12 (6.09) | 0 (0.00) |  |
| 2+ | 35 (15.62) | 32 (16.24) | 3 (11.11) |  |
| 3+ | 45 (20.09) | 40 (20.30) | 5 (18.52) |  |
| 4+ | 37 (16.52) | 30 (15.23) | 7 (25.93) |  |
| C1q |  |  |  | 0.198 |
| - | 214 (95.54) | 190 (96.45) | 24 (88.89) |  |
| + | 10 (4.46) | 7 (3.55) | 3 (11.11) |  |
| **Clinical parameters at biopsy** |  |  |  |  |
| Hypertension, *n*(%) | 74 (33.04) | 63 (31.98) | 11 (40.74) | 0.364 |
| Urinary RBCs (/μL) | 393.3 (69.92, 1767.58) | 427.3 (81.80, 1985.90) | 319.1 (62.20, 838.35) | 0.428 |
| Urinary WBCs (/μL) | 25.55 (8.95, 55.47) | 27.00 (10.00, 59.30) | 12.80 (3.55, 49.00) | 0.091 |
| Epithelial cells (/μL) | 6.75 (2.60, 14.77) | 6.70 (2.80, 14.60) | 6.80 (1.95, 16.80) | 0.669 |
| Tubular casts (/μL) | 0.85 (0.20, 1.50) | 0.80 (0.20, 1.50) | 0.90 (0.20, 1.30) | 0.835 |
| Hyaline casts (/μL) | 0.40 (0.10, 1.00) | 0.40 (0.10, 1.00) | 0.60 (0.10, 1.00) | 0.608 |
| Pathological casts (/μL) | 0.20 (0.00, 0.40) | 0.10 (0.00, 0.40) | 0.20 (0.05, 0.50) | 0.554 |
| β_2_-MG (mg/L) | 0.30 (0.20, 0.50) | 0.30 (0.20, 0.50) | 0.40 (0.20, 0.81) | 0.224 |
| BUN (μmol/L) | 4.86 (4.05, 6.12) | 4.82 (4.04, 6.10) | 5.60 (4.14, 6.16) | 0.39 |
| WBC (10^9^/L) | 7.38 (5.92, 9.26) | 7.40 (5.91, 9.26) | 7.26 (6.14, 9.43) | 0.928 |
| NEU (10^9^/L) | 3.96 (2.88, 5.91) | 3.93 (2.83, 5.94) | 4.22 (3.19, 5.72) | 0.654 |
| LYM (10^9^/L) | 2.50 (1.89, 3.13) | 2.50 (1.91, 3.17) | 2.32 (1.88, 2.92) | 0.292 |
| MONO (10^9^/L) | 0.44 (0.34, 0.62) | 0.44 (0.33, 0.62) | 0.42 (0.34, 0.54) | 0.655 |
| RBC (10^9^/L) | 4.40 (4.13, 4.71) | 4.39 (4.11, 4.71) | 4.49 (4.15, 4.74) | 0.471 |
| HGB (g/L) | 123 (114.00, 132.00) | 122 (114.00, 131.00) | 127 (119.50, 134.50) | 0.203 |
| PLT (10^9^/L) | 293 (242.25, 349.50) | 296 (247.00, 362.00) | 266 (219.50, 319.00) | 0.055 |
| NLR | 1.54 (1.05, 2.68) | 1.52 (1.04, 2.61) | 1.65 (1.29, 3.24) | 0.209 |
| LMR | 5.67 (4.04, 7.15) | 5.74 (3.91, 7.38) | 5.59 (4.30, 6.71) | 0.595 |
| PWR | 39.58 (31.20, 48.64) | 39.74 (31.22, 49.58) | 34.14 (30.17, 42.93) | 0.111 |
| PNR | 73.89 (48.79, 102.06) | 74.54 (49.52, 103.33) | 61.47 (44.45, 86.17) | 0.153 |
| PLR | 116.05 (93.63, 146.90) | 115.62 (93.69, 148.02) | 118.38 (94.92, 132.87) | 0.884 |
| PAR | 7.88 (6.11, 10.88) | 7.96 (6.29, 11.12) | 6.73 (5.68, 10.57) | 0.135 |
| ESR (mm/h) | 12.00 (6.00, 22.25) | 12.00 (6.00, 22.00) | 7.00 (4.50, 26.00) | 0.219 |
| TT (S) | 16.70 (16.20, 17.50) | 16.70 (16.20, 17.50) | 16.70 (16.35, 18.10) | 0.347 |
| PTA (%) | 97.5 (89.00, 108.00) | 98 (89.00, 108.00) | 96 (85.50, 106.00) | 0.492 |
| PT (S) | 13.30 (12.70, 13.90) | 13.30 (12.70, 13.80) | 13.50 (12.90, 14.10) | 0.361 |
| APTT (S) | 39.25 (35.98, 42.20) | 39.20 (36.00, 41.90) | 39.50 (36.05, 43.60) | 0.846 |
| FIB (g/L) | 3.42 (2.80, 4.08) | 3.46 (2.81, 4.10) | 3.19 (2.73, 3.84) | 0.355 |
| INR | 1.02 (0.96, 1.08) | 1.02 (0.96, 1.07) | 1.03 (0.96, 1.10) | 0.509 |
| FAR (mg/g) | 92.7 (68.00, 125.66) | 92.7 (68.00, 125.66) | 92.7 (68.00, 125.66) | 0.455 |
| ALT (U/L) | 13.00 (10.00, 18.00) | 13.00 (10.00, 18.00) | 13.00 (10.50, 17.50) | 0.757 |
| ALB (g/L) | 38.20 (30.75, 42.25) | 38.10 (30.60, 42.10) | 38.90 (32.90, 43.40) | 0.492 |
| GLB (g/L) | 26.45 (23.78, 29.30) | 26.50 (23.80, 29.30) | 25.10 (23.65, 29.20) | 0.466 |
| TBIL (μmol/L) | 4.40 (3.30, 6.20) | 4.40 (3.30, 6.10) | 5.10 (3.50, 6.75) | 0.536 |
| DBIL (μmol/L) | 1.60 (0.88, 2.00) | 1.60 (0.90, 1.90) | 1.60 (0.90, 2.65) | 0.142 |
| IBIL (μmol/L) | 3.15 (2.08, 4.23) | 3.20 (2.10, 4.20) | 3.10 (2.25, 4.40) | 0.88 |
| LDH (U/L) | 225 (192.75, 250.00) | 226 (194.00, 251.00) | 212 (188.50, 233.50) | 0.229 |
| TC (mmol/L) | 4.12 (3.45, 5.33) | 4.11 (3.45, 5.31) | 4.27 (3.46, 5.49) | 0.867 |
| TG (mmol/L) | 1.38 (0.86, 2.07) | 1.33 (0.86, 2.06) | 1.70 (1.05, 2.81) | 0.14 |
| HDL (mmol/L) | 1.23 (1.07, 1.52) | 1.25 (1.08, 1.56) | 1.15 (0.98, 1.35) | 0.163 |
| LDL (mmol/L) | 2.37 (1.88, 3.28) | 2.34 (1.90, 3.15) | 2.50 (1.75, 3.42) | 0.878 |
| ApoA (g/L) | 1.44 (1.23, 1.78) | 1.48 (1.23, 1.79) | 1.32 (1.17, 1.64) | 0.163 |
| ApoB (g/L) | 0.75 (0.58, 1.01) | 0.74 (0.58, 0.98) | 0.84 (0.48, 1.08) | 0.953 |
| ALP (U/L) | 180.88 ± 64.76 | 180.18 ± 61.15 | 185.93 ± 88.06 | 0.745 |
| Ca^2+^ (mmol/L) | 2.25 (2.16, 2.32) | 2.25 (2.16, 2.32) | 2.29 (2.17, 2.33) | 0.325 |
| 25(OH)D (ng/mL) | 12.30 (8.00, 17.10) | 12.50 (8.00, 17.30) | 11.60 (8.50, 15.80) | 0.561 |
| C3 (g/L) | 0.96 (0.85, 1.11) | 0.97 (0.85, 1.13) | 0.94 (0.87, 1.02) | 0.363 |
| C4 (g/L) | 0.20 (0.15, 0.25) | 0.20 (0.15, 0.25) | 0.20 (0.17, 0.25) | 0.743 |
| IgA (g/L) | 2.08 (1.44, 2.75) | 2.11 (1.47, 2.76) | 1.83 (1.29, 2.24) | 0.093 |
| IgE (IU/mL) | 58.11(20.40, 222.37) | 64.91(22.42, 227.70) | 33.72(14.41, 79.10) | 0.087 |
| IgG (g/L) | 8.10 (4.95, 10.10) | 8.10 (5.10, 10.00) | 8.70 (4.65, 10.80) | 0.502 |
| IgM (g/L) | 1.17 (0.93, 1.52) | 1.18 (0.93, 1.51) | 1.12 (0.92, 1.53) | 0.794 |
| C3/C4 | 4.82 (4.10, 6.22) | 4.84 (4.15, 6.23) | 4.71 (3.74, 5.68) | 0.274 |
| IgA/C3 | 2.09 (1.47, 2.95) | 2.10 (1.47, 3.06) | 1.90 (1.46, 2.50) | 0.243 |
| IgG/C3 | 7.61 (4.87, 10.96) | 7.59 (4.89, 10.91) | 9.88 (4.31, 11.64) | 0.341 |
| **Follow-up information** |  |  |  |  |
| Qualitative hematuria, *n*(%) |  |  |  | 0.649 |
| - | 141 (62.95) | 126 (63.96) | 15 (55.56) |  |
| ± | 38 (16.96) | 33 (16.75) | 5 (18.52) |  |
| 1+ | 23 (10.27) | 19 (9.64) | 4 (14.81) |  |
| 2+ | 16 (7.14) | 13 (6.60) | 3 (11.11) |  |
| 3+ | 6 (2.68) | 6 (3.05) | 0 (0.00) |  |
| Qualitative proteinuria, *n*(%) |  |  |  | 0.018 |
| - | 166 (74.11) | 151 (76.65) | 15 (55.56) |  |
| ± | 32 (14.29) | 28 (14.21) | 4 (14.81) |  |
| 1+ | 12 (5.36) | 9 (4.57) | 3 (11.11) |  |
| 2+ | 7 (3.12) | 5 (2.54) | 2 (7.41) |  |
| 3+ | 7 (3.12) | 4 (2.03) | 3 (11.11) |  |
| ALB at last follow-up (g/L) | 46.04 ± 2.87 | 46.08 ± 2.71 | 45.80 ± 3.90 |  |

Continuous variables are expressed as mean ± SD or median (IQR), and categorical variables as number (%). Based on whether poor progenies occurred, patients were divided into non-endpoint group and endpoint group. *IgA*, immunoglobulin A; *IgM*, immunoglobulin M; *IgG*, immunoglobulin G; *C3*, complement component 3; *C1q*, complement component 1q; *RBC*, red blood cell; *WBC*, white blood cell; *β2-MG*, beta-2 microglobulin; *BUN*, blood urea nitrogen; *NEU*, neutrophil count; *LYM*, lymphocyte count; *MONO*, monocyte count; *HGB*, hemoglobin; *PLT*, platelet count; *NLR*, neutrophil-to-lymphocyte ratio; *LMR*, lymphocyte-to-monocyte ratio; *PWR*, platelet-to-white cell ratio; *PNR*, platelet-to-neutrophil ratio; *PLR*, platelet-to-lymphocyte ratio; *PAR*, platelet-to-albumin ratio; *ESR*, erythrocyte sedimentation rate; *TT*, thrombin time; *PTA*, prothrombin activity; *PT*, prothrombin time; *APTT*, activated partial thromboplastin time; *FIB*, fibrinogen; *INR*, international normalized ratio; *FAR*, fibrinogen-to-albumin ratio; *AST*, aspartate aminotransferase; *ALT*, alanine aminotransferase; *ALB*, albumin; *GLB*, globulin; *TBIL*, total bilirubin; *DBIL*, direct bilirubin; *IBIL*, indirect bilirubin; *LDH*, lactate dehydrogenase; *TC*, total cholesterol; *TG*, triglycerides; *HDL*, high-density lipoprotein; *LDL*, low-density lipoprotein; *ApoA*, apolipoprotein A; *ApoB*, apolipoprotein B; *ALP*, alkaline phosphatase; *Ca²⁺*, calcium ion; *25(OH)D*, 25-hydroxyvitamin D; *C4*, complement component 4; *IgE*, immunoglobulin E.

**Supplementary Table 2 Screening of time-dependent predictors of eGFR slope**

| **Variable** | **Model** | **Overall eGFR slope** | | |  | **Chronic eGFR slope** | | |
| --- | --- | --- | --- | --- | --- | --- | --- | --- |
|  |  | **AIC** | **BIC** | **Chisq** |  | **AIC** | **BIC** | **Chisq** |
| Birth weight (kg) | 1 | 28726.75 | 28770.29 |  |  | 22499.48 | 22541.85 |  |
|  | 2 | 28725.56 | 28775.33 | 3.19 |  | 22499.52 | 22547.94 | 1.96 |
| Age at biopsy (years) | 1 | 28712.55 | 28756.09 |  |  | 22479.35 | 22521.71 |  |
|  | 2 | 28701.69 | 28751.45 | 12.86^***^ |  | 22471.7 | 22520.12 | 9.64^***^ |
| Oxford classification S score | 1 | 28716.48 | 28760.02 |  |  | 22490.43 | 22532.79 |  |
|  | 2 | 28717.62 | 28767.38 | 0.86 |  | 22491.93 | 22540.34 | 0.5 |
| Oxford classification E score | 1 | 28724.77 | 28768.32 |  |  | 22499.95 | 22542.32 |  |
|  | 2 | 28725.77 | 28775.54 | 1 |  | 22501.81 | 22550.23 | 0.14 |
| Gross hematuria status during follow-up | 1 | 28726.77 | 28776.53 |  |  | 22495.73 | 22538.1 |  |
|  | 2 | 28710.51 | 28772.72 | 20.25^***^ |  | 22497.33 | 22545.75 | 0.4 |
| Proteinuria remission status during follow-up | 1 | 28663.62 | 28713.39 |  |  | 22425.8 | 22474.22 |  |
|  | 2 | 28646.09 | 28708.29 | 21.54^***^ |  | 22412.54 | 22473.07 | 17.26^***^ |

Model 1 represents the baseline model, while Model 2 is the extended model incorporating interaction terms. Significant interaction (LRT-*p-value*<0.05 with lower AIC/BIC) indicated time-dependent effects. **p-value* <0.05; ***p-value* <0.01; ****p-value* <0.001. *eGFR*, estimated glomerular filtration rate; *AIC*, akaike information criterion; *BIC*, bayesian information criterion; *E*, endocapillary hypercellularity; *S*, segmental glomerulosclerosis.
